# Supplementary material for: Inflammatory factors and risk of lung adenocarcinoma: a Mendelian randomization study mediated by blood metabolites
Source: Front Endocrinol (Lausanne). 2024 Aug 27;15:1446863. doi: 10.3389/fendo.2024.1446863 (PMC11384989; doi:10.3389/fendo.2024.1446863)
Supplement: Supplementary file 1 [file DataSheet1.zip › supplementary files/S2/GCST90200768/sensitivity-analysis.pdf]

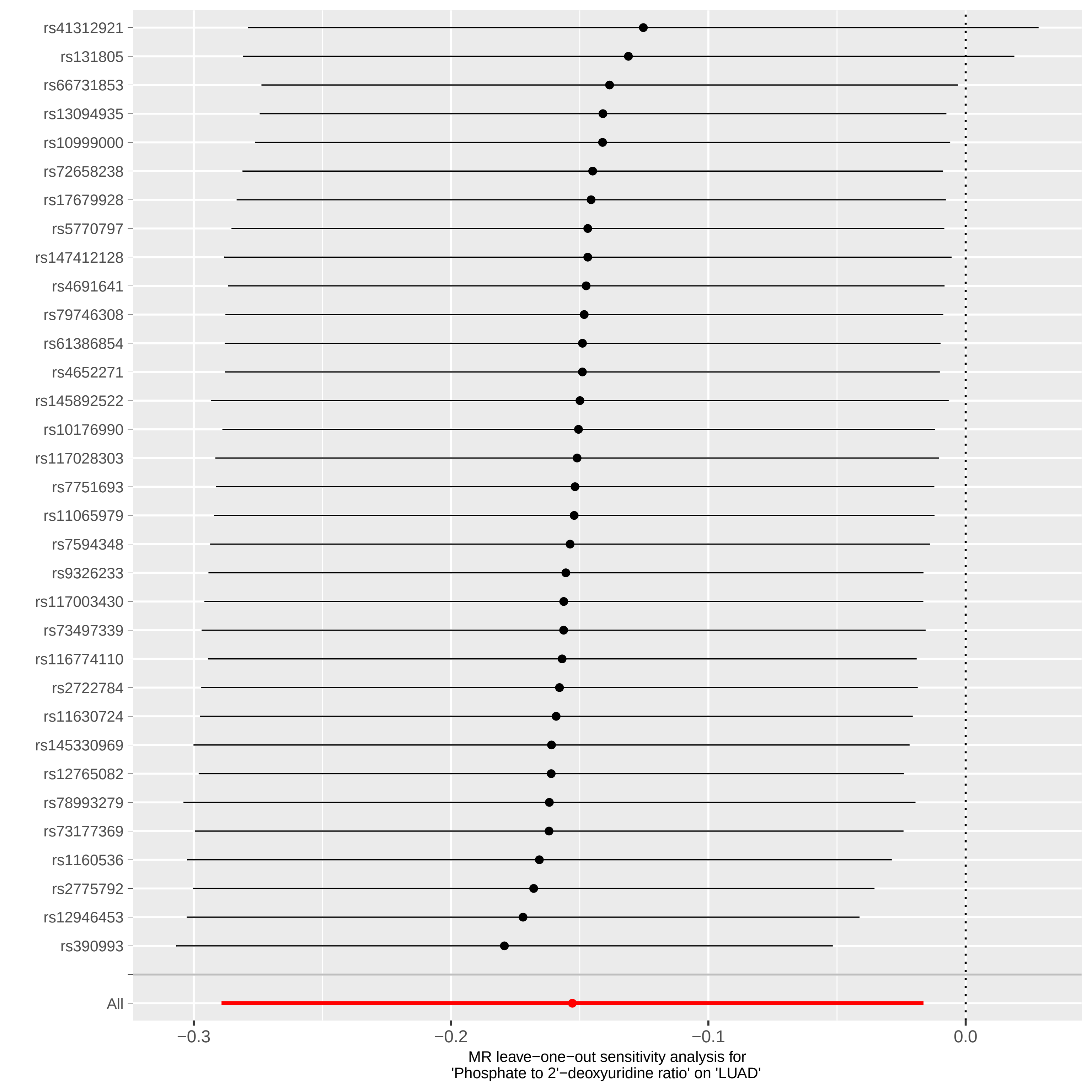
rs41312921
rs131805
rs66731853
rs13094935
rs10999000
rs72658238
rs17679928
rs5770797
rs147412128
rs4691641
rs79746308
rs61386854
rs4652271
rs145892522
rs10176990
rs117028303
rs7751693
rs11065979
rs7594348
rs9326233
rs117003430
rs73497339
rs116774110
rs2722784
rs11630724
rs145330969
rs12765082
rs78993279
rs73177369
rs1160536
rs2775792
rs12946453
rs390993
All
−0.3
−0.2
−0.1
0.0
MR leave−one−out sensitivity analysis for
'Phosphate to 2'−deoxyuridine ratio' on 'LUAD'
